# Functional Characterization of Circulating Mumps Viruses with Stop Codon Mutations in the Small Hydrophobic Protein

**DOI:** 10.1128/mSphere.00840-20

**Published:** 2020-11-18

**Authors:** Rita Czakó Stinnett, Andrew S. Beck, Elena N. Lopareva, Rebecca J. McNall, Donald R. Latner, Carole J. Hickman, Paul A. Rota, Bettina Bankamp

**Affiliations:** a Division of Viral Diseases, Centers for Disease Control and Prevention, Atlanta, Georgia, USA; b Oak Ridge Institute for Science and Education (ORISE), contracted to Division of Viral Diseases, Centers for Disease Control and Prevention, Oak Ridge, Tennessee, USA; University of Pittsburgh School of Medicine

**Keywords:** SH protein, genomics, molecular epidemiology, mumps virus, next-generation sequencing, paramyxovirus, surveillance studies, vaccine, vaccine preventable, altered termination, host-pathogen interactions

## Abstract

Between 2015 and 2017, routine molecular surveillance in the United States detected multiple mumps viruses (MuVs) with mutations in the small hydrophobic (SH) gene compared to a reference virus of the same genotype. These mutations include an unusual pattern of uracil-to-cytosine hypermutations and other mutations resulting in the generation of premature stop codons or disruption of the canonical stop codon. The mumps virus SH protein may serve as a virulence factor, based on evidence that it inhibits apoptosis and innate immune signaling *in vitro* and that recombinant viruses that do not express the SH protein are attenuated in an animal model. In this study, mumps viruses bearing variant SH sequences were isolated from contemporary outbreak samples to evaluate the impact of the observed mutations on SH protein function. All isolates with variant SH sequences replicated in interferon-competent cells with no evidence of attenuation. Furthermore, all SH-variant viruses retained the ability to abrogate induction of NF-κB-mediated innate immune signaling in infected cells. Ectopic expression of variant mumps SH genes is consistent with findings from infection experiments, indicating that the observed abrogation of signaling was not mediated by other viral factors that may modulate innate immune signaling. Molecular surveillance is an important public health tool for monitoring the diversity of circulating mumps viruses and can provide insights into determinants of disease. These findings, in turn, will inform studies employing reverse genetics to elucidate the specific mechanisms of MuV pathogenesis and potential impacts of observed sequence variants on infectivity, fitness, and virulence.

**IMPORTANCE** Mumps virus (MuV) outbreaks occur in the United States despite high coverage with measles, mumps, rubella (MMR) vaccine. Routine genotyping of laboratory-confirmed mumps cases has been practiced in the United States since 2006 to enhance mumps surveillance. This study reports the detection of unusual mutations in the small hydrophobic (SH) protein of contemporary laboratory-confirmed mumps cases and is the first to describe the impact of such mutations on SH protein function. These mutations are predicted to profoundly alter the amino acid sequence of the SH protein, which has been shown to antagonize host innate immune responses; however, they were neither associated with defects in virus replication nor attenuated protein function *in vitro*, consistent with detection in clinical specimens. A better understanding of the forces governing mumps virus sequence diversity and of the functional consequences of mutations in viral proteins is important for maintaining robust capacity for mumps detection and disease control.

## Abstract (precis)

Mumps virus (MuV) outbreaks occur in the United States despite high coverage with measles, mumps, rubella (MMR) vaccine. Routine genotyping of laboratory-confirmed mumps cases has been practiced in the United States since 2006 to enhance mumps surveillance. This study reports the detection of unusual mutations in the small hydrophobic (SH) protein of contemporary laboratory-confirmed mumps cases and is the first to describe the impact of such mutations on SH protein function. These mutations are predicted to profoundly alter the amino acid sequence of the SH protein, which has been shown to antagonize host innate immune responses; however, they were neither associated with defects in virus replication nor attenuated protein function *in vitro*, consistent with detection in clinical specimens. A better understanding of the forces governing mumps virus sequence diversity and of the functional consequences of mutations in viral proteins is important for maintaining robust capacity for mumps detection and disease control.

## OBSERVATION

Mumps is a highly contagious, vaccine-preventable viral illness that in the prevaccine era was associated with severe clinical outcomes, including deafness, encephalitis, and meningitis. The incidence of mumps in the United States decreased significantly after a vaccine became widely available, but mumps outbreaks continue to occur even among highly vaccinated populations (1). Mumps is an orthorubulavirus within the family *Paramyxoviridae* (2). The World Health Organization recognizes 12 distinct genotypes of MuVs based on diversity in the sequence of the gene coding for the small hydrophobic (SH) protein, which represents one of the least conserved regions of the single-stranded, negative-sense RNA genome of MuV (3). Since routine genotyping began in 2006, genotype G viruses have accounted for the majority of mumps cases in the United States (4, 5).

**Detection of noncanonical SH variants.** The Centers for Disease Control and the Association of Public Health Laboratories (APHL) established Vaccine Preventable Diseases Reference Centers, to enhance domestic mumps surveillance through genotyping of circulating MuVs (1, 3, 6). Phylogenetic analysis of contemporary MuV SH sequences from clinical samples submitted for laboratory testing between 2015 and 2017 detected several viruses in genotype G with unusual mutations. The SH sequences of these viruses were most closely related to the Sheffield reference strain for genotype G, MuVi/Sheffield.GBR/01.05 [G] (1).

**FIG 1:**
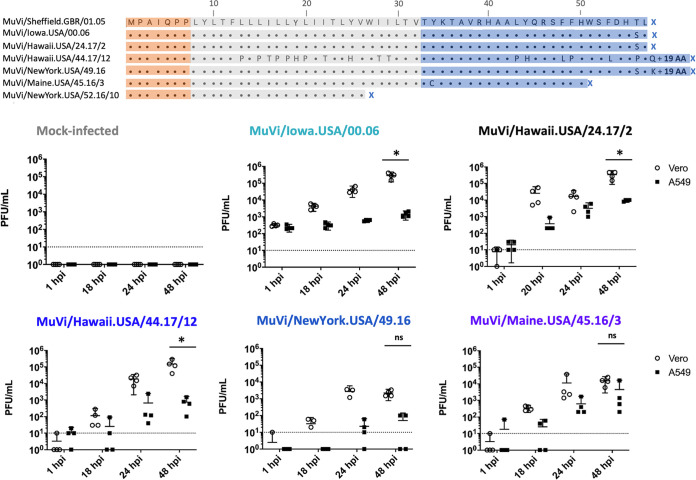
(A) Amino acid alignment of a panel of contemporary, noncanonical SH amino acid sequences aligned against the SH amino acid sequence of the reference virus MuVi/Sheffield.GBR/01.05[G]. MuV SH protein topology is indicated by the superimposed shaded boxes; the ectodomain, transmembrane domain, and cytoplasmic domain are shown in orange, gray, and blue, respectively. (B) The replication kinetics of virus isolates bearing variant SH genes were evaluated through paired experiments at an MOI of 0.1 in Vero cells, which have interferon signaling defects and are routinely used for MuV isolation, and in interferon-competent A549 cells. Supernatant was sampled at the indicated time points and infectious virus titers from four biological replicates of each condition were determined for all samples by plaque assay on Vero cells. The limit of detection is indicated by a dashed line. Results shown are representative of three independent experiments. The well-characterized isolate MuVi/Iowa.USA/00.06 bearing a canonical SH sequence was included for comparison to contemporary viruses.*, *P* < 0.05, Vero versus A549; ns, not significant (two-way ANOVA, by virus, with *post hoc* Sidak correction for multiple comparisons using GraphPad Prism v8).

Sequence alignment in MEGA (v10.0.4) revealed mutations predicted to alter SH protein termination, including substitutions that either replaced the standard stop codon to encode SH proteins with a predicted length of 76 amino acids (Fig. 1; MuVi/Hawaii.USA/44.17/12 and MuVi/NewYork.USA/49.16) or introduced premature stop codons to encode SH proteins with predicted lengths of 50 amino acids (Fig. 1; MuVi/Maine.USA/45.16/3) and 26 amino acids (Fig. 1; MuVi/NewYork.USA/52.16/10). Furthermore, the SH sequence of MuVi/Hawaii.USA/44.17/12 represents a cluster of 16 viruses with identical SH sequences that were reported throughout 2017; in addition to altered termination, these SH sequences displayed 26 U-to-C transitions, encoding 17 nonsynonymous substitutions.

Previous studies in a small animal model demonstrated that SH may contribute to mumps pathogenesis, likely through antagonism of innate immune responses by mechanisms that have been recently elucidated (7, 8). Here, we evaluated the impact of these naturally occurring mutations in the SH gene on the function of the MuV SH protein *in vitro*.

**Characterization of virus isolates.** Viruses listed in Fig. 1A were isolated from clinical samples and plaque purified on Vero cells (GenBank accession numbers for mumps SH sequences in Fig. 1: KF876699.1, JN012242, MF543822, MH654817, KY430355, KY322491, and KY764205, respectively). Infectious virus titers were determined by endpoint dilution in both A549 and Vero cells. Paired, multicycle growth kinetics experiments were performed in the interferon-competent A549 cell line and the interferon-deficient Vero cell line to evaluate virus replication (note: the stock titer of MuVi/NewYork.USA/52.16/10 was not sufficient to evaluate replication kinetics at a multiplicity of infection (MOI) of 0.1.) All evaluated isolates bearing mutations in SH sequence replicated to lower titers in A549 cells compared to Vero cells; however, similar attenuation of virus replication in A549 cells was observed in infections with a control virus bearing a canonical SH sequence (MuVi/Iowa.USA/00.06) and an outbreak isolate with no predicted amino acid substitutions in SH (MuVi/Hawaii.USA/24.17/2) (Fig. 1B). All viruses produced characteristic cytopathic effects of MuV infection, including formation of syncytia, following inoculation of Vero and A549 cells.

**FIG 2:**
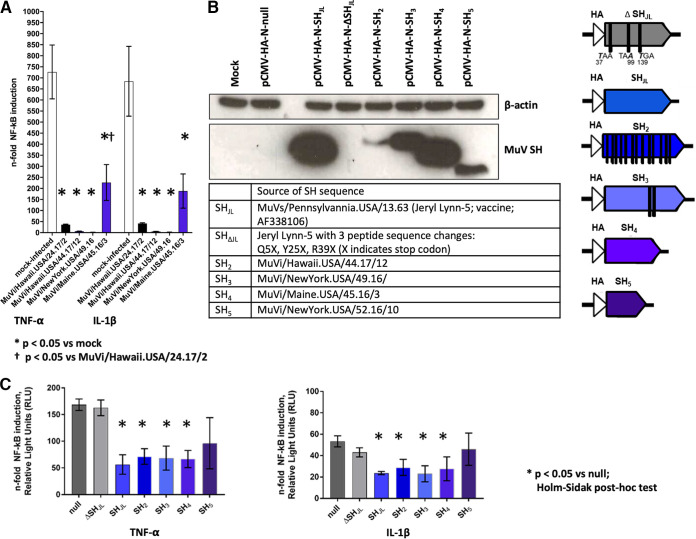
(A) A549 cells were transfected (X-tremeGENE HP DNA transfection reagent, Roche) with pCMV-β-Gal and p(PRDII)5tkΔ(−39)lucter, which carries a luciferase gene expressed under the control of an NF-κB-inducible promoter. Transfected A549 cells were inoculated at 24 h posttransfection with MuVs at an MOI of 0.5 and, 16 h later, were stimulated with either TNF-α or IL-1β or were not stimulated. Cell lysates were harvested 4 h poststimulation for quantification of luminescence and beta-galactosidase activity using commercially available assay systems (OneGlo Luciferase Assay System and Beta-Galactosidase Enzyme Assay System, respectively; Promega). Luminescence values from each sample were normalized based on beta-galactosidase activity and were expressed in relative light units (RLU). The fold change of NF-κB induction was estimated by calculating the ratio between RLU from stimulated versus unstimulated controls for each condition. The SH amino acid sequence of MuVi/Hawaii.USA/24.17/2 is identical to the reference strain MuVi/Sheffield/GBR/01.05 [G]; this condition was included as a positive control for SH-mediated abrogation of NF-κB signaling. Statistical analysis was performed by one-way ANOVA followed by *post hoc* Holm-Sidak correction for multiple comparisons in GraphPad Prism v8. (B) Noncanonical SH sequences were cloned for ectopic expression through high-fidelity assembly (NEBuilder HiFi Assembly Mix; New England BioLabs) into expression vector pCMV-HA-N. Plasmid constructs were verified by Sanger sequencing (BigDye v3.1, ABI). A549 cells were transfected with the indicated plasmids (X-tremeGENE HP DNA transfection reagent, Sigma-Aldrich) and lysates were harvested at 24 h posttransfection. Expression of N-terminally tagged SH proteins was confirmed by immunoblot following SDS-PAGE of lysates (20 μg total protein) in denaturing conditions (12% Bis-Tris protein gel in MES buffer, Invitrogen) and transfer to nitrocellulose using the iBlot system (Invitrogen). Membranes were probed for HA (H3663, Sigma-Aldrich) and endogenous β-actin (A3854, Sigma-Aldrich), which served as a loading control. Sources and characteristics of the cloned SH sequences are summarized in text and in graphic form, respectively. (C) A549 cells were cotransfected with reporter plasmids and either null vector or SH expression vectors, as described in panel A. Cells were stimulated with either TNF-α or IL-1β at 24 h posttransfection and then harvested for detection of luminescence and beta-galactosidase activity.

A luciferase-based reporter assay system was used to probe the ability of MuVs bearing SH variants to antagonize innate immune signaling. A549 cells were transfected with a reporter construct in which luciferase expression was regulated by an NF-κB-inducible promoter element with a beta-galactosidase construct used as a transfection control. Transfected A549 cells were inoculated with MuVs (MOI = 0.5) and, 16 h later, were stimulated with tumor necrosis factor alpha (TNF-α) or interleukin 1 beta (IL-1β) and harvested for quantification of luminescence, as previously described (8). Infection with MuVi/Hawaii.USA/24.17/2, which bears a canonical SH sequence, was included as a positive control for MuV infection-mediated inhibition of NF-κB upregulation. Infection with contemporary MuVs bearing noncanonical SH sequences each influenced induction of NF-κB-mediated signaling *in vitro* to various degrees; however, in each case, we observed evidence of statistically significant inhibition compared to the mock condition (Fig. 2A).

**Functional studies of ectopically expressed SH protein variants.** To rule out the contribution of other MuV genes in the observed inhibition of NF-κB signaling, noncanonical SH sequences were cloned into expression vector pCMV-HA-N for ectopic expression of N-terminally HA-tagged SH proteins. No commercial antibodies are available for the detection of MuV SH proteins. Expression of N-terminally tagged SH proteins was confirmed by immunoblot of transfected A549 cell lysates. A construct bearing the SH sequence of mumps vaccine strain Jeryl Lynn (SH_JL_), and a previously described mutant with 3 stop codons introduced to abrogate SH protein expression (SH_ΔJL_) (9), were included as positive and negative controls for SH activity, respectively. Tagged SH protein was detected under all conditions except the mock transfection, null expression vector, and SH_ΔJL_ controls (Fig. 2B). Detection of tagged SH protein in the remaining conditions corresponded with the expected molecular weights of the SH_JL_ protein and the four noncanonical SH proteins (Fig. 2B).

A549 cells were cotransfected with reporter plasmids and either the null vector or a MuV SH construct, as described above. Each condition was evaluated in triplicate, and luciferase signal was normalized by beta-galactosidase activity. Again, abrogation of NF-κB-mediated signaling was expressed as the ratio of normalized relative light units (RLU) for stimulated versus unstimulated cells for each condition. Results shown are representative of three independent experiments (Fig. 2C). Fold change induction in luciferase activity was indistinguishable between the null vector and the SH_ΔJL_ negative-control conditions. In contrast, expression of noncanonical SH sequences was associated with statistically significant inhibition of NF-κB-mediated signaling under all conditions but one, confirming the contribution of SH to the observed inhibition of NF-κB-mediated signaling in the context of MuV infection. Notably, inhibition mediated by the shortest SH protein, MuVi/New York.USA/52.16/10, was not statistically different from SH_ΔJL_.

**FIG 3:**
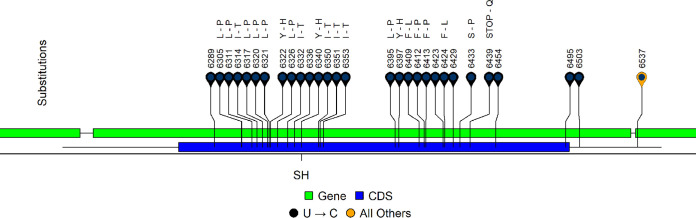
SH substitutions observed in seven sequenced MuV strains bearing hypervariable SH genes, relative to MuVi/Iowa.USA/00.06. Strains were geographically and temporally similar to MuVi/Hawaii.USA/44.17/12, the source of noncanonical SH_2_ in Fig. 2B. All sequenced isolates shared the same substitutions in this region of the MuV genome. Substitutions were classified as synonymous or coding (amino acid substitution is noted); U-to-C substitutions are noted by color. For instances where multiple substitutions contribute to the coding result at a single codon, they were considered separately for all reported calculations.

**Whole-genome sequencing of MuV strains encoding noncanonical SH.** To better understand the genomic context of the noncanonical SH sequences, whole-genome sequencing by stranded Illumina chemistry was performed on MuVi/Hawaii.USA/44.17/12 (source of SH_2_ in Fig. 2B) and six other isolates with the same hypermutated SH gene (Fig. S1 in the supplemental material). The seven isolates were from geographically and temporally related cases spanning epidemiologic weeks 23 through 44 of 2017 (Table S1). N/S ratios were greatest along the SH protein (3.0, all other proteins were ≤0.65) (Fig. S2a). Although mutations were detected in each transcriptional unit of the MuV genome, whole-genome sequences showed evidence of hypermutation only in SH (Fig. 3, Fig. S2b). This mutational pattern in SH was shared across all seven sequenced isolates; all substitutions (*n* = 28) observed in the SH gene were U-to-C transitions, including a TAA(Stop)/CAA(glutamine) substitution extending the predicted SH coding DNA sequence (CDS) from 57 to 76 amino acids in length (Fig. 3). This mutational signature is characteristic of editing by the host enzyme adenosine deaminase acting on RNA 1 (hADAR1) (10–15). The algorithm InosinePredict (16) was used to probe the SH sequence of MuVi/Sheffield.GBR/01.05 [G] for predicted hADAR1 editing sites; 30/32 (94%) mutations occurred at a predicted editing site (Fig. S3). The assembly pipeline is described in the methods section of the supplemental material (Text S1), with coverage statistics described in Table S2.

10.1128/mSphere.00840-20.1 TEXT S1 Supplementary methods for next-generation sequencing pipeline used to assemble consensus sequences for MuV strains encoding noncanonical SH genes. Download Text S1, DOCX file, 0.01 MB. Copyright © 2020 Stinnett et al. 2020 Stinnett et al. This content is distributed under the terms of the Creative Commons Attribution 4.0 International license.

10.1128/mSphere.00840-20.4 FIG S1 Assembly of Illumina sequencing for MuV strains encoding noncanonical SH genes (MuVi/Hawaii.USA/44.17/12 and six others with identical SH sequences), showing read coverages relative to an annotated whole-genome sequence of a genotype G virus, MuVi/Iowa.USA/00.06 (JN012242). Assemblies were complete in all cases, meaning that all viral genes were covered. Coverage statistics for the assemblies are shown in Table S2. Download FIG S1, TIF file, 0.7 MB. Copyright © 2020 Stinnett et al. 2020 Stinnett et al. This content is distributed under the terms of the Creative Commons Attribution 4.0 International license.

10.1128/mSphere.00840-20.5 FIG S2 Summary of mutations in the seven MuV strains sequenced by Illumina methods. From the assembled consensus, the mutations were classified against an annotated genotype G reference, MuVi/Iowa.USA/00.06. The total count of substitutions observed in the seven sequences was 131, of which 91 were synonymous and 40 coded for amino acid substitutions (N/S ratio = 0.44). Tabulation of classified substitutions supports the presence of hypervariable nonsynonymous substitutions biased toward the SH coding region. (A) Nonsynonymous/synonymous ratios by gene, expressed as the mean N/S ratio for each sequence (*n* = 7); this was done to account for slight sequence differences outside SH. Greater N/S ratios for the SH gene are observed in comparison to other viral genes. V/P gene substitutions were tabulated separately with respect to each of the V/P/I protein coding frames, which are differentiated by the presence of an RNA editing tract. (B) Normalized counts of all substitutions per viral gene, expressed as a ratio of total substitution counts to gene length in bases. Chart columns express the mean ratio accounting separately for each sequence (*n* = 7); this was done to account for slight sequence differences between the sequenced isolates. Higher normalized counts were observed in the SH gene relative to the other viral genes, reflecting a mutational bias in SH. (C) Proportion of substitution types observed in all seven sequenced isolates. The total number of substitutions was 131 relative to MuVi/Iowa.USA/00.06. U-to-C transitions predominate in the observed set, comprising 48% of all mutations observed. Download FIG S2, TIF file, 0.4 MB. Copyright © 2020 Stinnett et al. 2020 Stinnett et al. This content is distributed under the terms of the Creative Commons Attribution 4.0 International license.

10.1128/mSphere.00840-20.6 FIG S3 The algorithm InosinePredict (16) contains an empirically derived model predicting the intensity of hADAR1 adenosine-to-inosine editing at a particular RNA base while considering neighboring sequence content; this was used to probe the SH sequence of MuVi or s/Sheffield/GBR/01.05[G] for the presence of hADAR1 substrate motifs. Vertical bars (graphic) indicate modeled inference of editing by hADAR1 at the indicated base, as expressed by a normalized percentage of A-to-I reaction product expected in a synthetic control RNA species, under standardized reaction conditions. Blue boxes indicate positions of observed mutations in hypermutated SH sequences. Download FIG S3, TIF file, 0.4 MB. Copyright © 2020 Stinnett et al. 2020 Stinnett et al. This content is distributed under the terms of the Creative Commons Attribution 4.0 International license.

10.1128/mSphere.00840-20.2 TABLE S1 Description of strains sequenced in the study by Illumina methods. Whole-genome sequences were deposited to Genbank using the WHO-recommended nomenclature “MuVi” in the strain name, indicating production of the sequence from a cell culture isolate; sequence read archive (SRA) accessions contain refined BAM alignments that were used to guide final consensus base calls. Accession numbers for the original SH genotyping of clinical specimens (indicated using the WHO-recommended nomenclature “MuVs”) are listed in the rightmost column. Download Table S1, XLSX file, 0.01 MB. Copyright © 2020 Stinnett et al. 2020 Stinnett et al. This content is distributed under the terms of the Creative Commons Attribution 4.0 International license.

10.1128/mSphere.00840-20.3 TABLE S2 Coverage statistics showing the median and standard deviation (SD) of reads aligning to individual bases in the assembly, listed by the whole genome as aggregate (leftmost data column) and by individual genes. Plots of read coverage for the assemblies are shown in Fig. S1. Download Table S2, XLSX file, 0.01 MB. Copyright © 2020 Stinnett et al. 2020 Stinnett et al. This content is distributed under the terms of the Creative Commons Attribution 4.0 International license.

**Discussion.** The relative frequency by which variant SH sequences arise in circulation, as well as the overall genetic diversity of mumps genome diversity, are difficult to estimate in the absence of widespread molecular surveillance. Nevertheless, MuV SH sequences with altered termination and hypermutation have previously been reported in different regions of the world, and have been identified in samples from multiple patients as early as 2004 (5, 17). The unusual SH sequences described by Jin et al. (5) and Cui et al. (17) were derived from genotype G and genotype C viruses and also display patterns of either U-to-C or C-to-U transitions predicting altered SH protein termination. Most recently, McNall et al. described sequences from genotype K viruses that predicted a SH protein of 71 amino acids (1). The observation of these shared and independently occurring patterns of alteration in SH sequences across time and space in multiple genotypes suggests that these mutations do not have obvious negative impacts on viral fitness; however, the forces influencing the emergence of these mutations are not clear. The genomes of circulating paramyxoviruses appear to be relatively stable. Intragenotypic diversity in SH has been estimated to be less than 20% (5), and the substitution rate of the SH gene has been estimated to be less than 2 × 10^−2^ substitutions/site/year (17) and less than 1 × 10^−3^ substitutions/site/year across the MuV genome (18, 19). Nevertheless, serial *in vitro* passage experiments demonstrate that the MuV genome can accumulate clusters of stable mutations in a nonuniform fashion across the genome within a relatively low number of passages (20).

The role of the SH protein in MuV infection is not clear. Paramyxoviruses express multiple antagonists of host innate immunity, including the MuV SH protein and its homologs from related paramyxoviruses (21). This role is supported by observations that SH protein expression is associated with impaired induction of inflammatory cytokines and antagonism of NF-κB-mediated proapoptotic programs *in vitro*, as well as attenuation in an animal model (7, 8). However, the introduction of stop codons in the SH sequence of infectious molecular clones was not associated with attenuation in a rat model of mumps neurovirulence (9), suggesting that the correlation between SH sequence and function is not straightforward. Furthermore, replication experiments in interferon-competent and interferon-deficient cells in this study showed no unique defects for viruses with noncanonical SH sequences compared to viruses with canonical SH sequences, consistent with previous observations that the SH protein is dispensable for virus replication (7, 9, 22).

Hypermutation may reflect the relative dispensability of the mutated gene product for viral fitness in the physiological setting. Biased U-to-C hypermutation in paramyxoviruses was first described for the measles matrix (M) gene and is a characteristic feature of viral sequences recovered from cases of subacute sclerosing panencephalitis and measles inclusion body encephalitis. It has been proposed that these mutations are acquired over the course of persistent replication in the brain, which leads to the selection of mutants with defects in the M protein and glycoproteins. (23, 24). A similar pattern of biased U-to-C hypermutation has been described in the context of mumps vaccine virus genomes recovered from the brain of a previously vaccinated pediatric severe combined immunodeficiency (SCID) patient. Consensus hypermutation in the M gene was observed in this strain alongside limited quasispecies diversity by next-generation sequencing, suggesting either (i) absence of negative selective pressures acting on divergent M sequences or (ii) positive selection of defective M protein as a means to achieve efficient viral spread in nervous tissue (25). In support of the first mechanism, a similar pattern of biased U-to-C hypermutation in the SH sequence has been observed in nonprimate strains of parainfluenza virus 5, accompanied by mutation of the start codon and loss of SH protein expression (26). Furthermore, a study contrasting sequences of MuV strains from parotitis-only cases versus specimens from cases with neurological complications reported no clear association between observed amino acid substitutions in SH and disease phenotype (27).

The pattern of U-to-C mutations observed in this study is consistent with the editing signature of hADAR1. Editing of viral transcripts by this host enzyme has been demonstrated in the context of measles infection *in vitro* and has also been proposed to be responsible for sequence differences between two mumps vaccine strains (10–15). In contrast, the altered termination in the absence of U-to-C hypermutation likely arose through an independent mechanism. The C-terminal domain of the mumps SH protein is more variable than the N-terminal ectodomain (8). Together with the findings reported here, this suggests that the critical determinants of SH function may be found in the conserved N-terminal ectodomain. Future studies will be required to identify the domains necessary and sufficient for SH function.

This study had several limitations. The presence of an N-terminal HA tag may have influenced the folding, expression level, and subcellular localization of ectopically expressed SH proteins. Impacts on SH protein function also cannot be ruled out, although the activity of tagged SH_JL_ and SH_ΔJL_ in this study was consistent with previous observations. The levels of ectopically expressed SH protein may also not be representative of physiological levels of SH protein expression in the context of virus replication, which may influence the outcome of interactions with host factors. Future studies that investigate the impact of the observed mutations on SH protein expression and localization through production of recombinant viruses via a reverse genetics system are necessary. Furthermore, the consequences of the observed alterations in SH proteins on viral fitness and transmission in a physiological setting are difficult to predict. The clinical features, vaccination status, and nature of the epidemiological relationship between cases from which these sequences were derived are unknown.

Our findings suggest that the function of the MuV SH protein may be less sequence-dependent than previously recognized. This idea is supported by the observation that several paramyxoviruses encode SH proteins with similar anti-apoptotic activity but little sequence homology (21). Furthermore, recombinant SV5, a closely related rubulavirus, engineered to express the MuV SH open reading frame (ORF) instead of its native homolog, showed *in vitro* anti-apoptotic activity comparable to wild-type SV5 (28). Ultimately, the mechanisms by which these mutations arise in the MuV genome and their contributions to pathogenesis require further examination. The growing database of publicly available MuV sequences is an important resource that presents opportunities to inform functional studies to identify key molecular determinants of MuV pathogenesis.

**Data availability.** Consensus sequences and Illumina reads were respectively deposited to GenBank and SRA under BioProject PRJNA322324. GenBank accession numbers are listed in Table S1 in the supplemental material for genomes sequenced in this study, and throughout the text for previously submitted sequences.
