# Supplementary material for: Functional Characterization of Circulating Mumps Viruses with Stop Codon Mutations in the Small Hydrophobic Protein
Source: mSphere. 2020 Nov 18;5(6):e00840-20. doi: 10.1128/mSphere.00840-20 (PMC7677008; doi:10.1128/mSphere.00840-20)
Supplement: TEXT S1 [file mSphere.00840-20-s0001.docx]

## Text S1

## Supplemental Methods

## Whole genome sequencing of MuV strains encoding non-canonical SH.

MuV isolates were passaged on Vero cells as indicated (Table S1), and RNA from supernatant virus was column-purified from Trizol-LS using the Direct-Zol® kit (Zymo Research, Irvine, CA). Mammalian rRNA was removed from 50 ng of viral RNA, with subsequent DNAse digestion, using NEBNext® depletion reagents (NEB, Ipswich, MA). Stranded libraries were generated using NEBNext® RNA reagents (NEB). Libraries were sequenced on a MiSeq® instrument using paired-end chemistry (2 x 150 cycle) (Illumina, San Diego, CA).

## Sequencing Analysis Pipeline

Reads were trimmed of adaptor and low-quality base content using Trimmomatic v.0.36, and then locally aligned to reference JN012242 (MuVi/Iowa.USA/00.06[G]) using Bowtie v.2.3.3.1 (very-sensitive presets in all use cases) in the absence of a whole genome reference for the genotype G reference virus MuVi/Sheffield.GBR/01.05. A hybrid genome-guided *de novo* assembly was performed on these alignments using Trinity v.2.1.1, from which the resulting contigs were aligned and oriented to JN012242 using MAFFT v.7.313; alignment was agnostic of contig strand orientation (adjust-direction). A consensus assembly was generated by simple majority from the contig alignment using the “cons” utility of Emboss v.6.5.7 (-plurality 2). Reads were remapped to the *de novo* contig consensus using Bowtie v.2.3.3.1. PCR duplicates were removed using the MarkDuplicates utility of PicardTools v.2.5.0. Alignment accuracy was refined using a single pass of the IndelRealigner utility of GATK v.3.6. Final, refined read sets were imported to Geneious v.11.1.4, and consensus sequences were generated by simple majority rule. In this environment, final consensus sequences were mapped again to JN012242; substitutions were classified in reference to this mapping, and annotations were likewise transferred for public database upload. Pipeline was controlled and documented using Snakemake v.5.5.4 and is available from the authors for review upon request. Consensus sequences and sequencing reads were respectively deposited to Genbank and SRA under BioProject PRJNA322324.
