# Supplementary figures and images for: Functional Characterization of Circulating Mumps Viruses with Stop Codon Mutations in the Small Hydrophobic Protein
Source: mSphere. 2020 Nov 18;5(6):e00840-20. doi: 10.1128/mSphere.00840-20 (PMC7677008; doi:10.1128/mSphere.00840-20)

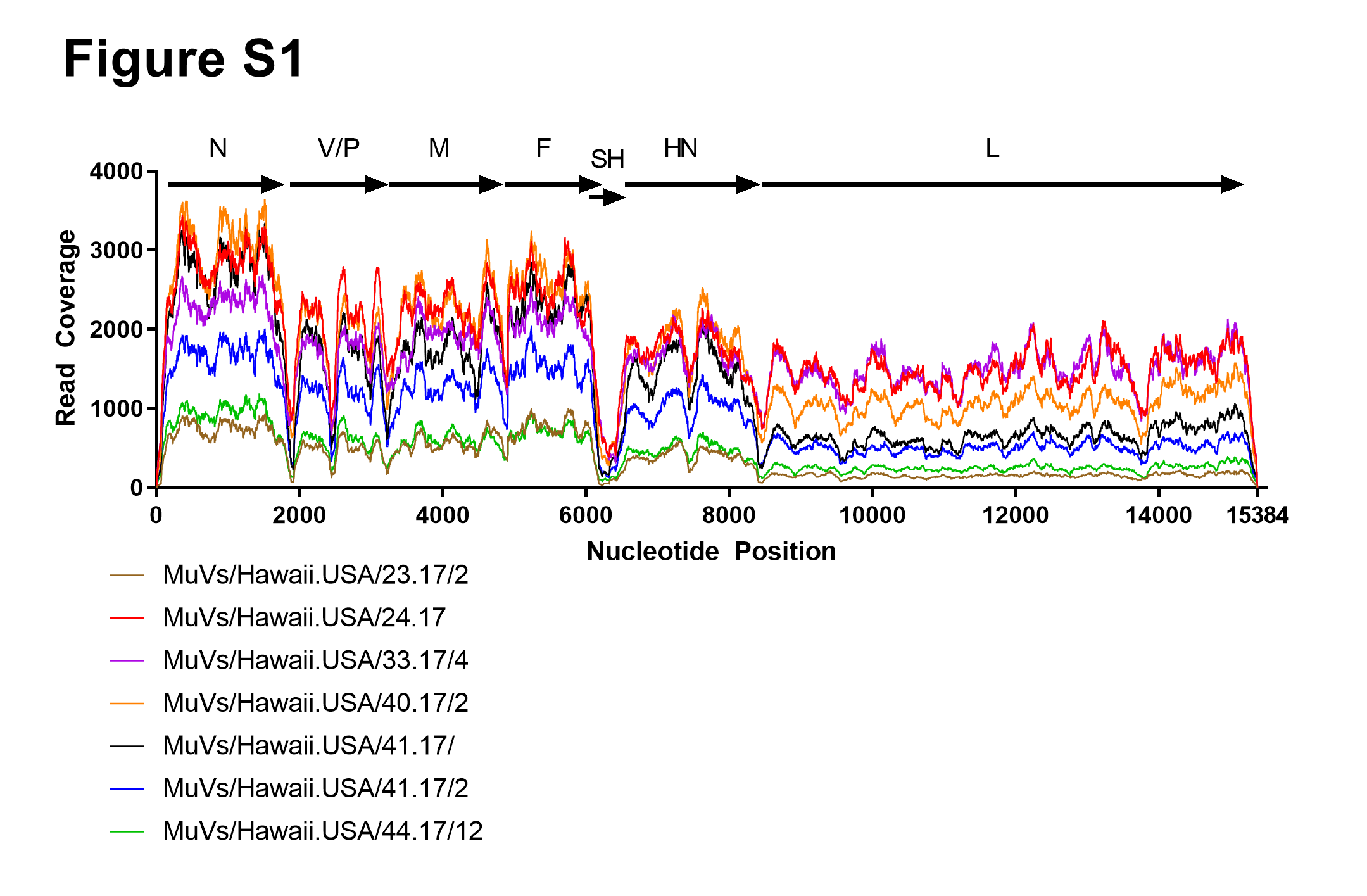

Supplement: FIG S1 [file mSphere.00840-20-sf001.tif]

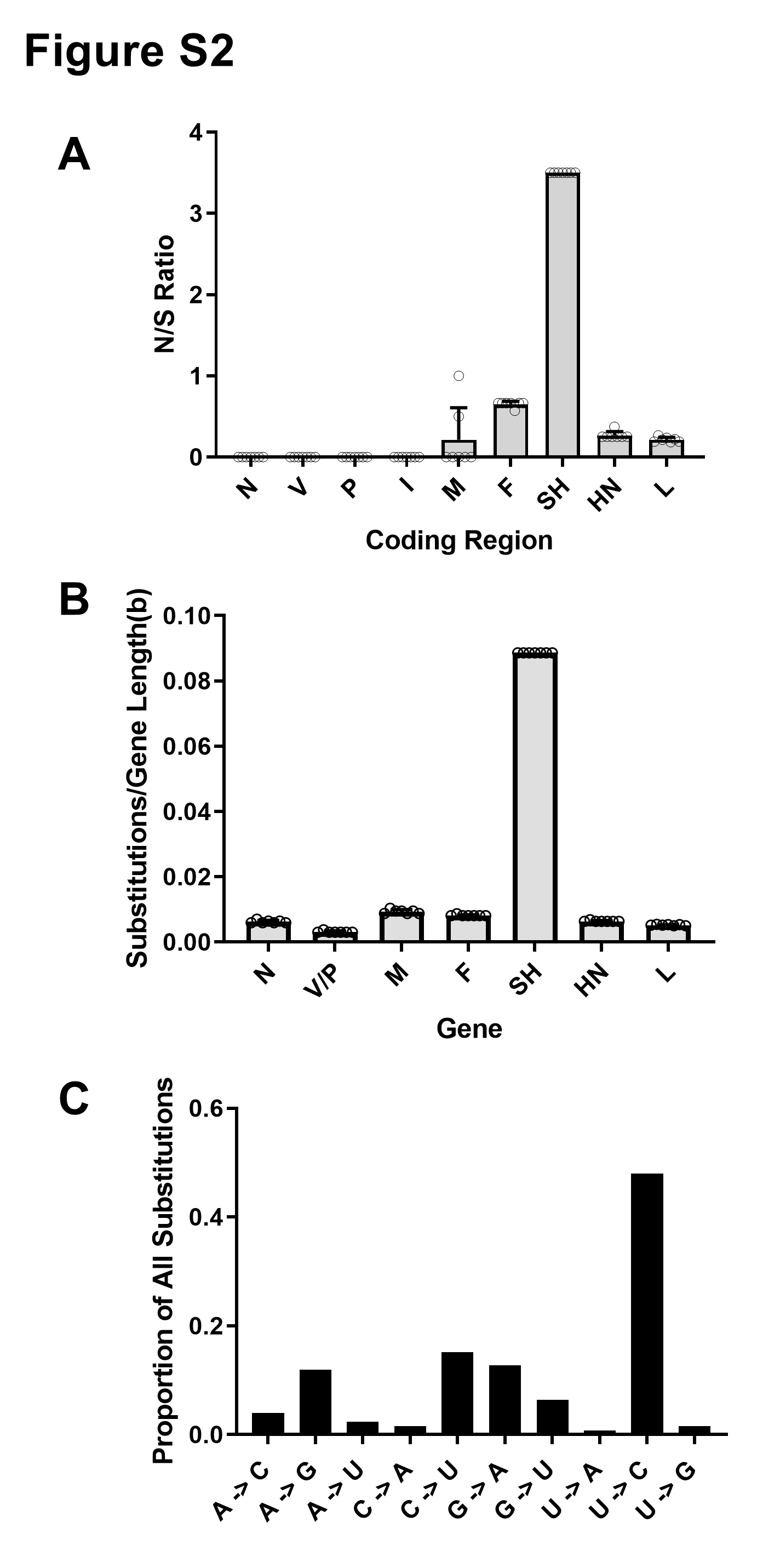

Supplement: FIG S2 [file mSphere.00840-20-sf002.tif]

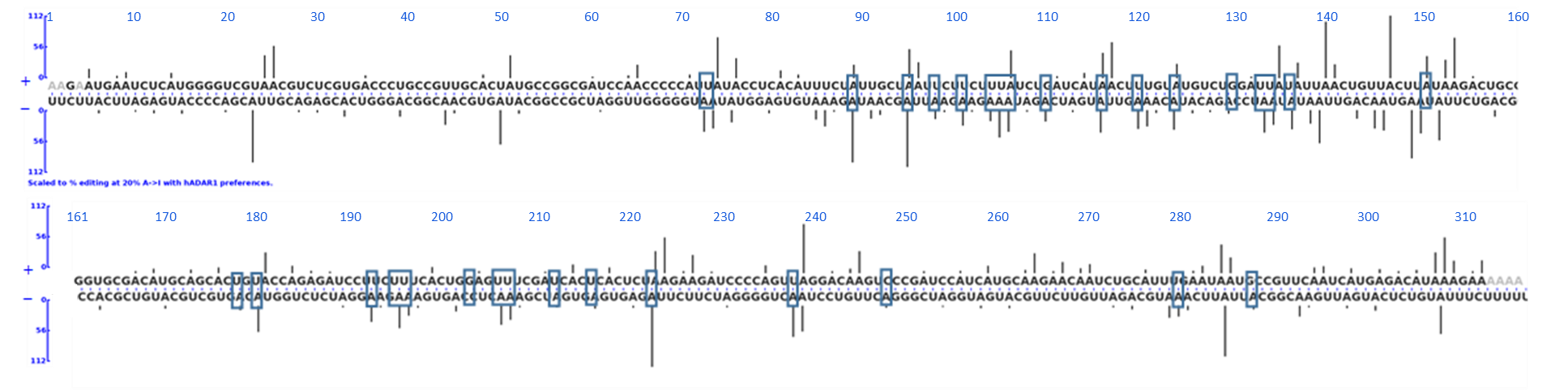

Supplement: FIG S3 [file mSphere.00840-20-sf003.tif]
